# Evaluation of unmet clinical needs in prophylaxis and treatment of venous thromboembolism in at-risk patient groups: pregnancy, elderly and obese patients

**DOI:** 10.1186/s12959-019-0214-8

**Published:** 2019-12-27

**Authors:** Benjamin Brenner, Roopen Arya, Jan Beyer-Westendorf, James Douketis, Russell Hull, Ismail Elalamy, Davide Imberti, Zhenguo Zhai

**Affiliations:** 1 Department of Hematology and Bone Marrow Transplantation, Rambam Health Care Campus, Haifa, Israel; 2 Department of Obstetrics and Gynaecology, The First I.M. Sechenov Moscow State Medical University, Moscow, Russia; 3 King’s Thrombosis Centre, Department of Haematological Medicine, King’s College Hospital Foundation NHS Trust, London, UK; 4 Thrombosis Research Unit, Department of Medicine I, Division Hematology, University Hospital ‘Carl Gustav Carus’ Dresden, Dresden, Germany; 5 King’s Thrombosis Service, Department of Haematology, King’s College London, London, UK; 6 Department of Medicine, McMaster University, Hamilton, Ontario Canada; 7 Thrombosis and Atherosclerosis Research Institute, Hamilton, Ontario Canada; 8 Foothills Medical Centre and Thrombosis Research Unit, University of Calgary, Calgary, Canada; 9 Hematology and Thrombosis Center, Tenon University Hospital, Sorbonne University, INSERM U938, Sorbonne University, Paris, France; 10 Hospital of Piacenza, Piacenza, Italy; 11 Department of Pulmonary and Critical Care Medicine, Center of Respiratory Medicine, China-Japan Friendship Hospital, National Clinical Research Center for Respiratory Diseases, Beijing, China

**Keywords:** Venous thromboembolism, Elderly, Pregnant, Pregnancy, Obese, Obesity, Anticoagulants, Low-molecular-weight heparin

## Abstract

**Background:**

Venous thromboembolism (VTE) accounts for an estimated 900,000 cases per year in the US alone and constitutes a considerable burden on healthcare systems across the globe.

**Objective:**

To understand why the burden is so high, qualitative and quantitative research was carried out to gain insights from experts, guidelines and published studies on the unmet clinical needs and therapeutic strategies in VTE prevention and treatment in three populations identified as being at increased risk of VTE and in whom VTE prevention and treatment were regarded as suboptimal: pregnant women, the elderly and obese patients.

**Methodology:**

A gap analysis methodology was created to highlight unmet needs in VTE management and to discover the patient populations considered most at risk. A questionnaire was devised to guide qualitative interviews with 44 thrombosis and haemostasis experts, and a review of the literature on VTE in the specific patient groups from 2015 to 2017 was completed. This was followed by a Think Tank meeting where the results from the research were discussed.

**Results:**

This review highlights the insights gained and examines in detail the unmet needs with regard to VTE risk-assessment tools, biomarkers, patient stratification methods, and anticoagulant and dosing regimens in pregnant women, the elderly and obese patients.

**Conclusions:**

Specifically, in pregnant women at high risk of VTE, low-molecular-weight heparin (LMWH) is the therapy of choice, but it remains unclear how to use anticoagulants when VTE risk is intermediate. In elderly patients, evaluation of the benefit of VTE prophylaxis against the bleeding risk is particularly important, and a head-to-head comparison of efficacy and safety of LMWH versus direct oral anticoagulants is needed. Finally, in obese patients, lack of guidance on anticoagulant dose adjustment to body weight has emerged as a major obstacle in effective prophylaxis and treatment of VTE.

## Background

Venous thromboembolism (VTE), comprising deep vein thrombosis (DVT) and pulmonary embolism (PE), remains a major concern for healthcare systems globally. Despite improved prophylaxis and treatment options, and current risk-assessment tools, morbidity and mortality related to VTE remains high in patient populations such as pregnant women, the elderly and obese patients [1].

VTE is the main cause of mortality in women during the post-partum period [2]. Acute VTE is linked to substantial long-term mortality in the elderly (21% of 991 patients in a Swiss cohort study with a median follow-up time of 30 months) [3]. Patients with VTE who are morbidly obese are more likely to have extended hospital and intensive care unit stays [4]. Thus, the suboptimal use of anticoagulants in these patients and the increased cost burden related to longer hospital stays needs to be addressed.

A lack of adequate population-specific risk-assessment tools, along with uncertainties around correct dosing regimens and concern over increased bleeding risk, may be linked to these elevated mortality and morbidity rates [5, 6]. In addition, although guidelines exist on prophylaxis and treatment of these patients, discrepancies occur between recommendations, leading to low adherence by physicians to such guidance [7–9]. Moreover, there is a paucity of evidence on these patient populations due to problems of recruiting individuals to randomised controlled trials (RCT), which may be linked to patients’ comorbidities, frailty and concern over foetal development and maternal well-being [10, 11].

Considering the above, it is important to examine the evidence presented in guidelines, published studies and reviews, and through expert opinion, in order to highlight unmet needs and inconsistencies in clinical practice, with a view to homogenising VTE prevention and treatment strategies.

## Methodology

Quantitative mapping was performed to identify key opinion leaders who were active online and in publications, patient advocacy groups, scientific associations, editorial boards, guidelines, clinical trials and congress activities in the thrombosis and haemostasis field. From this list, experts were selected from a range of countries dependent on their availability to attend a telephone interview. Forty-four key opinion leaders were contacted between February and August 2017 from 12 different countries or regions: Canada, Brazil, five European countries, Middle East, Africa, Russia, China and Japan. The interviews followed a pre-determined questionnaire [see Additional file 1] and a gap analysis was carried out on the information received. The data revealed areas of unmet need with regard to VTE management with cancer, the critically ill, pregnant women, the elderly and obese patients. Of these five patient groups, the first two were discussed in a previous paper and the latter three were chosen for discussion in this paper [12] additional file 2. A comprehensive literature search was conducted in PubMed, Cochrane Library and current guidelines (January 2015 to December 2018) using the terms: pregnant, pregnancy, obese, obesity, elderly and venous thromboembolism. Further insights were gained during a Thrombosis Think Tank meeting in Paris in February 2018, during which the authors discussed the findings from the qualitative and quantitative research in order to establish unmet clinical needs and examine therapeutic approaches to bridging the gaps in VTE management in these three patient groups.

### Prophylaxis and treatment of VTE during pregnancy and post-partum

Despite a relatively low absolute risk of VTE of 1.2 per 1000 pregnancies, VTE remains a leading cause of maternal mortality in developed countries [2, 13, 14]. VTE can occur at any time during pregnancy, but increases 20-fold during the post-partum period [13]. Timely diagnosis depends on awareness of the condition and recognition of risk factors, including a family history of or previous thrombophilia (heritable: antithrombin deficiency, protein C deficiency, protein S deficiency, factor V Leiden, prothrombin gene mutation; acquired: antiphospholipid antibodies, persistent lupus anticoagulant and/or persistent moderate/high titre anticardiolipin antibodies and/or β2-glycoprotein 1 antibodies) or VTE, obesity, increased maternal age, reduced mobility and hospitalisation, and is critical to avoid VTE-induced mortality [13].

#### Guideline recommendations for pregnant and post-natal women at risk of VTE

Various guidelines on prevention and treatment of VTE in ante- and post-partum women and women with recurrent pregnancy loss exist, and of the experts interviewed, the American College of Chest Physicians (ACCP) and the Royal College of Obstetricians and Gynaecologists (RCOG) were indicated as the main guidelines being followed (Table 1).

**Table 1:** Guidelines followed by experts interviewed

Question	Expert opinion
What guidelines and clinical protocols do you use for prevention and treatment of VTE, including guidance on dose and duration, in ante- or post-partum pregnant women and in women with recurrent pregnancy loss?	• ACCP/CHEST
	• ISTH
	• Italian Society of Thrombosis and Haemostasis
	• RCOG
	• National guidelines
	• Involved in the generation of national guidelines
	• Follow own experience
	• No guidelines are being followed

ACCP/CHEST, American College of Chest Physicians; ISTH, International Society of Thrombosis and Haemostasis; RCOG, Royal College of Obstetricians and Gynaecologists; VTE, venous thromboembolism

However, due to a lack of evidence-based data in this population, the recommendations provided by national and international guidelines often vary and have not recently been updated, apart from the American Society of Hematology (ASH) guidelines on VTE in pregnancy published in 2018 [10, 14]. For example, the guidelines on prophylaxis of VTE in women after a caesarean section show divergent recommendations. A study by Palmerola, et al., comparing guideline recommendations for thromboprophylaxis after a caesarean section from RCOG, the American College of Obstetricians and Gynecologists, and ACCP found that 85, 1 and 35% of patients, respectively, would receive pharmacologic prophylaxis if the guidance were followed, thus highlighting significant gaps in consistency between recommendations [15].

The underlying cause is the lack of RCTs in this patient population and over-reliance on observational data, especially case–control studies that provide a lower level of evidence [16]. Many studies on prevention and management of VTE in pregnancy are performed on a small patient population due to patient enrolment difficulties, as women are reluctant to take additional medication, particularly when it is administered through injections. For example, the TIPPS study aimed to examine the effects of dalteparin in pregnancy and recruited only 292 pregnant women with thrombophilia over 12 years [17]. Thrombophilia was defined in this study as two abnormal tests and no normal tests for protein S, protein C or antithrombin; two positive tests for anticardiolipin immunoglobulin M (IgM) (> 30 U/ml), anticardiolipin immunoglobulin G (IgG) (> 30 U/ml), anti-β2 glycoprotein IgG (> 20 U/ml), anti-β2 glycoprotein IgM (> 20 U/ml), or lupus anticoagulant; and one positive test for factor V Leiden (heterozygous or homozygous) or prothrombin gene defect (heterozygous or homozygous) [17]. Most of the studies involving pregnant women provide outcomes without achieving statistical significance and, due to an absence of high-level evidence, prophylaxis is often not provided [16]. Insights from the qualitative research carried out for this paper noted that in China, country-level guidelines have not yet been developed and there is an inconsistent approach to prophylaxis.

#### VTE risk-assessment models and biomarkers

A history of VTE or heritable thrombophilia (factor V Leiden mutation, prothrombin gene mutation, antithrombin deficiency, protein C deficiency, protein S deficiency) are established risk factors of VTE in pregnant women [18]; however, the data from the qualitative interviews underlined the need to develop new tools to identify additional risk factors for pregnant women. The STRATHEGE score study by Chauleur, et al., involving pregnant women with at least one VTE risk factor, established a simple scoring system to evaluate VTE risk, but the low event rate meant the discriminatory power of the score could not be assessed [19]. However, a subsequent study, aimed at evaluating the effectiveness of the STRATHEGE score following its implementation in 21 French maternity units, demonstrated a significantly reduced risk of VTE and placental vascular complications of 50 and 30%, respectively [20]. Another VTE risk score, which was developed through a logistic regression model and based on 14 risk factors, including comorbidities and VTE history in the first 6 weeks post-partum, offers a benefit of predicting VTE events in the early post-partum period more accurately than current models provided by UK and Swedish national guidelines, but further validation is needed [21]. Alternatively, the EThIG trial assessed a risk evaluation strategy and effectiveness of heparin prophylaxis in low-risk and high/very high-risk pregnant women groups. Risk-stratified dalteparin prophylaxis was associated with a low incidence of symptomatic VTE and few adverse events [22].

In terms of biomarkers, it is known that D-dimer levels, an exclusion criterion for VTE, increase during pregnancy and peak in the third trimester at levels above the conventional cut-off, making them of little use [23]. Several studies have looked at recording D-dimer reference intervals during the three trimesters in healthy pregnancy and suggested pregnancy-associated cut-off levels that may assist clinical decision-making on VTE prophylaxis [23, 24]. Soluble fibrin monomer forms a complex with fibrinogen in the bloodstream early in coagulation, and measuring levels of the complex has also been proposed as a marker to screen for VTE [25]. However, recent studies have questioned the predictive utility of all conventional and candidate VTE biomarkers for use during pregnancy and the puerperium [26, 27].

In summary, further research is needed to develop more precise risk-assessment tools and improve the diagnostic value of biomarkers in order to tailor thromboprophylaxis for this patient population.

#### Prophylaxis and treatment of VTE in ante- and post-partum periods

Thromboprophylaxis is recommended in all pregnant women with an estimated VTE risk above 5% but is advised against for a risk below 1%. However, the approach to the management of pregnant women with an estimated risk between 1 and 5% remains debatable (Table 2) [2, 14, 30].

**Table 2:** Subpopulations of pregnant women recommended for LMWH prophylaxis or treatment

Question	Expert opinion	Guideline recommendations
Which subpopulation(s) of pregnant women, ante- or post-partum, or those with recurrent pregnancy loss, should be treated with LMWHs such as enoxaparin?	• Women with recurrent pregnancy loss • No evidence to support use of LMWH to prevent recurrent pregnancy loss • Women with antiphospholipid syndrome or with heterozygosity of factor V Leiden mutation • Those undergoing IVF • Those with previous unprovoked or provoked VTE • LMWH is recommended in the case of a severe event such as placenta abruption, intrauterine foetus death or VTE	• ACCP/CHEST [28]: For women requiring long-term VKA treatment who are attempting pregnancy, a switch to LMWH is recommended. In women with no VTE risk factors, prophylaxis is not recommended following a caesarean section. No routine prophylaxis for patients following assisted reproduction • ASH [14]: Prophylaxis is only advised for women undergoing assisted reproductive therapy with severe ovarian hyperstimulation syndrome. For women with previous unprovoked or provoked VTE, ante-partum prophylaxis is advised. For women with antithrombin deficiency who are homozygous for the factor V Leiden regardless of family history, ante-partum and post-partum prophylaxis is recommended. In those with protein S or C deficiency, post-partum prophylaxis is advised • Italian Society of Thrombosis and Haemostasis [29]: Ante- and post-partum prophylaxis is recommended for women with thrombophilic defects. LMWH is recommended in women with prior VTE. Ante- and post-partum LMWH prophylaxis is suggested for women with prior obstetric complications and one thrombophilic defect • RCOG [13]: LMWH is the preferred anticoagulant to treat acute VTE and for antenatal and post-natal prophylaxis. 10 days prophylaxis with LMWH is recommended after an emergency caesarean section and after a planned caesarean section if there are additional risk factors

ACCP/CHEST, American College of Chest Physicians; ASH, American Society of Hematology; IVF, in-vitro fertilisation; LMWH, low-molecular-weight heparin; RCOG, Royal College of Obstetricians and Gynaecologists; VKA, vitamin K antagonists; VTE, venous thromboembolism

The 2015 RCOG guidelines state that prophylaxis should be used from the start of pregnancy in women with four VTE risk factors, from week 28 in those with three risk factors, and women with two risk factors should receive 10 days of post-partum prophylaxis [13]. This implies that nearly half of pregnant women are eligible for post-partum prophylaxis [31]. The ACCP guidelines suggest that the presence of one of the major risk factors or two minor risk factors, or one following emergency caesarean section indicates a post-partum VTE risk > 3% [28].

In the post-partum period, the risk of VTE is high in the first 2 weeks after giving birth. Guidelines [13, 28] suggest that prophylaxis should continue for 6 weeks post-partum, although experts noted that, considering the increase in risk is greatest in the first 2–3 weeks only, this recommendation may be contested, unless a history of VTE is present in a patient. Since VTE risk is high in the first week following a caesarean section, thromboprophylaxis is given post-partum for 10 days in the UK following all non-elective caesarean sections and for elective caesarean-section patients with one other VTE risk according to RCOG guidance [13]. This may account for the observed decrease in maternal deaths from 1985 to 2014 [32]. In Germany, post-partum prophylaxis depends on the type of caesarean section, i.e., prophylaxis after elective caesarean section lasts for 10–14 days and after an emergency caesarean section is extended for up to 3 months.

Direct oral anticoagulants (DOAC) should not be used in pregnancy, or when breastfeeding, as their effects on the foetus or the new-born child are currently unknown [14, 33, 34]. However, despite guideline recommendations, clinical experience in Germany (as documented subjectively by local opinion leaders) and a recent review of 137 pregnant women with DOAC exposure suggest that administration of DOACs during early pregnancy does not indicate a high risk of embryopathy, and pregnancy termination for these women may not be necessary [35].

In summary, low-molecular-weight heparin (LMWH) is the preferred anticoagulant for both prophylaxis and treatment during pregnancy. However, guidelines and opinions differ on how to stratify risk, the most effective duration of prophylaxis and the safety of DOACs during pregnancy.

#### LMWH dose adjustment

In pregnant women at very high risk of thromboembolic complications and especially in those with acute VTE, monitoring of anti-Xa activity is often recommended (and performed), aiming to ensure adequate dosing of LMWH, which can be challenging in pregnant women. However, there is considerable uncertainty about the strategy (peak or trough anti-Xa levels) and the target ranges, the impact of these target ranges on clinical outcomes, and the accuracy and reproducibility of the assays [36]. Taken together, the experts agreed that anti-Xa monitoring in pregnant women at very high risk for thromboembolism is widely used and likely beneficial, but also agreed that many details of this strategy are still under debate, indicating a large unmet need for better evidence in this setting (Table 3).

**Table 3:** Methods of identifying optimal anticoagulant dose in thrombophilic pregnant women and those with pregnancy loss

Question	Expert opinion	Guideline recommendations
What method do you use to identify optimal dose of anticoagulants in thrombophilic pregnant women and those with pregnancy loss, e.g., PK/PD modelling or other methods?	• Anti-Xa monitoring • Factor Xa activity in prophylaxis is not measured • Routine monitoring of the dose is not recommended, the clinical picture of each patients is more important • PK/PD data is not usually used • The PK/PD profile is required • LMWH dose adjusted to weight • Fixed dose • Full-dose enoxaparin for high-risk patients	• ACCP/CHEST [28]: Anti-Xa measuring is not advised. Intermediate-dose LMWH dose is recommended in pregnant women with a history of VTE, with thrombophilia or with a risk of pregnancy loss • ASH [14]: Routine anti-Xa monitoring to guide dosing is not advised • Italian Society of Thrombosis and Haemostasis [29]: Monitoring platelet count during prophylaxis with LMWH is advised. No evidence to suggest use of anti-Xa monitoring to adjust LMWH dose • RCOG [13]: Titration of LMWH dose against the woman’s booking or early pregnancy weight is advised. Routine measurement of anti-Xa is not recommended except in women < 50 kg or > 90 kg

ACCP/CHEST, American College of Chest Physicians; ASH, American Society of Hematology; LMWH, low-molecular-weight heparin; PK/PD, pharmacokinetic/pharmacodynamic; RCOG, Royal College of Obstetricians and Gynaecologists; VTE, venous thromboembolism

Clinical practice in the UK recommends dose adjustment as per body weight in pregnant women for both treatment and prophylaxis, and, as a result, only a few breakthrough clots occur, although generous dosage given as recommended by RCOG guidelines may be a reason for these outcomes [13]. In Israel, anti-Xa levels are measured for both treatment and prophylaxis, although usually approximately 60% of women on a therapeutic dose and 20% of women on a prophylactic dose need these doses to be adjusted at around 20–25 weeks. In Italy, the experts interviewed used a fixed dose of LMWH for prophylaxis in pregnant women with a history of thrombosis.

Some of the experts have questioned the ideal dose of LMWH for thromboprophylaxis and treatment in pregnancy. The ongoing Highlow study, an RCT of intermediate-dose LMWH adjusted to actual body weight versus fixed low-dose nadroparin, may inform this clinical question for thromboprophylaxis [37].

### Challenges in the management of VTE in elderly patients

With life expectancy increasing in the developed world, a new definition of ‘*the elderly’* should be considered, which should include significant comorbidities such as coronary, hepatic, renal and cognitive functions, as well as frailty, rather than focusing on age alone (Table 4).

**Table 4:** Practical considerations for treating elderly patients with high risk of VTE

Question	Expert opinion	Guideline recommendations
Are there any practical considerations when treating elderly patients with high risk of VTE, such as specific risk factors, contra-indications, comorbidities or practicalities of administration?	• Higher bleeding risk • Traditional regimens increase the risk of bleeding • The risk of internal bleeding • Need to evaluate the risk of stroke through bleeding • Renal function may be compromised • Dosage due to the reduction in kidney function • Dosage taking into consideration contra-indications • Co-medications • Lack of clinical trials • Affordability is an issue	All recommendations are non-age specific. ACCP/CHEST [28]: • Hepatic failure, severe renal failure, rheumatic disease, current cancer and age ≥ 80 are all independent risk factors for bleeding NICE [38]: • Balance the patient’s risk of VTE against their bleeding risk SIGN [39]: • Patients undergoing total hip replacement with increased risk of bleeding should be given mechanical prophylaxis alone

ACCP/CHEST, American College of Chest Physicians; NICE, The National Institute for Health and Care Excellence; SIGN, Scottish Intercollegiate Guidelines Network; VTE, venous thromboembolism

The interviewed experts noted that impaired renal and cognitive functions, but not age per se, may be the major factors influencing the decision for or against antithrombotic therapy, as well as treatment outcome. However, 26 of the interviewees acknowledged that such patients are usually excluded from clinical trials, which limits evidence and guideline recommendations [40]. Evidence shows that the risk of venous thrombosis, which associates with illnesses characteristic to advanced age, increases exponentially with age, but thromboprophylaxis remains suboptimal in this patient group due to fear of bleeding since thrombotic and bleeding risk profiles usually overlap in this population [41, 42].

#### VTE risk-assessment models and biomarkers in the elderly

The experts agreed that VTE risk assessment in elderly patients should include comorbidities, concomitant medications and frailty to identify those at high risk of VTE. Furthermore, biomarkers may help to increase the predictive performance of VTE risk-assessment strategies. In the setting of primary VTE prophylaxis in acutely ill medical patients, the MAGELLAN study found that in patients with an average age of 71.4 years, high concentrations of D-dimer (> 2 μg mL^− 1^ mean) at day 10 were a predictor of increased VTE risk for up to 35 days [43]. Subsequently, this informed the selection criteria for the APEX study, which used a D-dimer level of ≥2x the upper limit of normal to examine primary VTE prevention for acutely ill medical patients aged 60–74 [44]. The ADJUST-PE study demonstrated that an age-adjusted D-dimer cut-off of age × 10 in patients > 50 years was successful in ruling out patients at risk of VTE [45]. However, using this biomarker to drive primary VTE prophylaxis decisions may not be effective in elderly patients due to an increase of circulating D-dimer in this patient population, which may not necessarily be linked to increased VTE risk [42, 46]. Consequently, further studies need to establish a more accurate threshold for biomarkers such as D-dimer before they can be routinely used for risk stratification and treatment decisions.

At the same time, elderly fragile patients are at an increased risk of falls and bleeding, but bleeding scores are unreliable in this population and their use is limited [47]. Moreover, elderly women seem to be at 20–25% higher risk of bleeding than men [11, 48, 49].

The experts noted that VTE risk-assessment guidance differs across countries. Further work is needed to develop a simple-to-use risk-assessment score for elderly patients that incorporates age, gender, comorbidities and bleeding risk.

#### Consideration of anticoagulants for prophylaxis in the elderly

There is little evidence regarding ideal anticoagulants for prophylaxis of the elderly largely due to the under-representation of this group of patients in clinical studies, owing to several comorbidities which increase the chance of exclusion from a trial [40]. Therefore, certain guideline recommendations may have been extrapolated from studies with younger cohorts and may not necessarily extend to this patient population [42]. Patients > 75 years of age have an increased risk of VTE [42] and, according to the ACCP, hospitalised medical patients > 70 years should be offered pharmacological VTE prophylaxis with fondaparinux, LMWH or unfractionated heparin (UFH) [29]. Yet, a systematic review and meta-analysis comparing efficacy and safety of LMWH versus UFH reported an overall increase in the rate of major haemorrhage with heparin prophylaxis compared to no prophylaxis [50]. However, the LMWH group showed a statistically significant bleeding risk reduction over the UFH group and LMWH demonstrated a better efficacy profile than UFH in terms of reducing DVT risk (Table 5) [50].

**Table 5:** Subgroups of elderly patients for whom LMWH may be the optimal choice

Question	Expert opinion	Guideline recommendations
In which subgroups of elderly patients would you consider LMWHs, such as enoxaparin, the optimal choice?	• Only if the patient has a specific condition • In patients with cancer and VTE • In patients with ACS • Used in percutaneous coronary interventions, ACS and thrombolytic therapy • Those with a history of internal bleeding • LMWH preferred due to the ability to change dosage based on kidney function and age • Intermediate risk PE • Patients with acute PE who do not use DOACs • Patients with comorbidities, GI problems and chronic inflammatory disease • Patients with provoked VTE post-operatively • LMWH used with inpatients but not used with outpatients	All recommendations are non-age specific. ACCP/CHEST [28]: • Acutely ill hospitalised patients at increased risk of thrombosis • Critically ill patients • Outpatients with solid tumours who have additional risk factors for VTE and low bleeding risk NICE [38]: • Patients with renal impairment needing pharmacological VTE prophylaxis • People with myeloma or pancreatic cancer receiving chemotherapy • People receiving palliative care • Those admitted to the critical care unit • 1 month of VTE prophylaxis for patients with fragility fractures of the pelvis, hip or proximal femur • 10 days of LMWH for people undergoing elective hip replacement surgery • 7 days minimum VTE prophylaxis with LMWH for patient undergoing open vascular surgery or major endovascular procedures, lower limb amputation SIGN [39]: • Patients undergoing total hip replacement should receive prophylaxis with LMWH • Patients with cancer and cancer surgery • In patients with non-haemorrhagic stroke at high risk of VTE • Patients with suspected PE or DVT should receive therapeutic doses

ACCP/CHEST, American College of Chest Physicians; ACS, acute coronary syndrome; DOAC, direct oral anticoagulant; DVT, deep vein thrombosis; GI, gastrointestinal; LMWH, low-molecular-weight heparin; NICE, The National Institute for Health and Care Excellence; PE, pulmonary embolism; SIGN, Scottish Intercollegiate Guidelines Network; VTE, venous thromboembolism

The experts agreed that thromboprophylaxis should only be prescribed following careful benefit–risk assessments, but it is essential to consider drug compliance, major and non-major bleeding risks, and comorbidities, including renal function, hypertension, infections and coronary artery disease. Evidence from expert interviews demonstrates disparities in thromboprophylaxis practice from country to country. In general, elderly patients are underprophylaxed due to the perceived increased risk of bleeding in this population [51]. In Germany, evidence from a VTE registry shows that patients > 65 years of age are often underprophylaxed out of hospital and increasing public awareness on VTE risk situations has been suggested as a possible solution [52]. In France, according to national experts, elderly patients often receive prophylaxis but many of these patients are prophylaxed with either the incorrect type of anticoagulant or a suboptimal dose, therefore increasing the bleeding risk without achieving antithrombotic effect [53]. In China, in-hospital prophylaxis is insufficient and a lack of VTE knowledge and understanding of the guidelines is leading to non-standard approaches to thromboprophylaxis [54]. Published data from Italian national registries have shown that in contrast to widely used extended prophylaxis following a surgical procedure, medical prophylaxis is rare [55].

Expert opinion suggests that extended prophylaxis of 35 days should be given for patients at high risk of VTE, such as post-surgery or cancer patients (Table 6).

**Table 6:** Extended prophylaxis in elderly patients

Question	Expert opinion	Guideline recommendations
Should extended prophylaxis be used in elderly patients, e.g., for hip fractures?	• In patients with cancer • In patients undergoing surgery • Hip/knee replacements • In patients with multiple fractures at risk of recurrent VTE • Injections can only be used for 2 weeks, oral is the preferred treatment • Generally given for 10–14 days but can be extended to 30–35 days • Primary prophylaxis is currently recommended for 35 days • Recommended for 1 month but often extended for 3 months • This should only be for very high-risk patients but we don’t know how to identify them • Yes, but length of time is not well defined	All recommendations are non-age specific. ACCP/CHEST [28]: • Extended-duration thromboprophylaxis up to 35 days reduces VTE in hip replacement, hip fracture and abdominal malignancy surgery NICE [38]: • There is a recommendation for research by the NICE guideline committee regarding extended-duration prophylaxis for patients undergoing elective total hip replacement surgery SIGN [39]: • Following total hip replacement, particularly those with previous VTE

ACCP/CHEST, American College of Chest Physicians; NICE, The National Institute for Health and Care Excellence; SIGN, Scottish Intercollegiate Guidelines Network; VTE, venous thromboembolism.

This statement was supported by Dentali, et al., 2016, who conducted a pooled analysis that suggested a potential benefit of extended antithrombotic prophylaxis in acutely ill patients [56]. Indeed, the EXCLAIM trial showed reduced rates of VTE in medically ill patients > 75 years of age when LMWH was administered for up to 38 days following hospital discharge; however, this was counterbalanced by an increase in major bleeding [57]. Therefore, extended prophylaxis should be assessed on an individual basis [58].

#### VTE treatment and secondary prevention after VTE in elderly patients

Elderly patients receiving therapeutic anticoagulation for VTE are at increased risk of long-term mortality with comorbid burden, polypharmacy and a low level of physical activity as predictors of major bleeding, one of the most common causes of death [3]. Thus, selection of the optimal antithrombotic agent, its dose and duration of treatment, and whether this should continue beyond hospitalisation is particularly important. Although LMWH remains an option for hospitalised, elderly patients with different comorbidities such as chronic inflammatory conditions, gastrointestinal system problems, poor renal function, active cancer, acute or chronic lung or heart conditions, and acute infections, extended-duration treatment with LMWH is rare, mainly due to its inconvenience and cost [59, 60]. As a consequence, short courses of initial LMWH therapy are followed by oral anticoagulation with a vitamin K antagonists (VKA) such as warfarin. During the last decade, DOACs have taken the place of LMWH/VKAs in acute and long-term VTE treatment due to the convenience of administration and an excellent dose-response relationship without the need for monitoring or frequent dose adjustments [61]. Indeed, several reviews examining study evidence on DOACs compared to VKAs for VTE treatment in patients ≥75 years of age have demonstrated better efficacy and safety of DOACs over VKAs, with no increase in the risk of bleeding [62, 63]. However, when the different DOACs are examined individually, varying profiles are revealed. The AMPLIFY trial noted improved efficacy and safety of apixaban (10 mg twice daily for 7 days followed by 5 mg twice daily for 6 months) compared to standard therapy (enoxaparin followed by warfarin) in patients ≥75 years [64], whereas the RE-COVER II trial, which compared dabigatran to warfarin, found that efficacy and risk of bleeding increased exponentially with age with dabigatran and decreased with warfarin [65]. The Hokusai-VTE study, comparing edoxaban to warfarin, observed an increased bleeding risk linked to age regardless of treatment, but noted a reduction in recurrent VTE for patients > 80 years of age on the DOAC regimen [66]. In the EINSTEIN-DVT and PE trials, there was a 1.4% reduction in recurrent VTE events and a 3.3% reduction in major bleeding in patients ≥75 years of age receiving rivaroxaban compared to standard enoxaparin/VKA treatment [67].

The elderly subpopulations of patients ≥75 years of age in these trials, however, consist of small sample sizes from 13 to 43% of the total study populations [63]. In addition, the majority of these trials compared DOACs to VKAs, and clinical trials are now needed to directly compare extended treatment with LMWHs to treatment with DOACs in elderly patients ≥75 years of age, along with a need for real-life evidence in elderly patients with a high risk of DOAC accumulation [62].

In summary, more studies need to be carried out to establish the most effective and safe antithrombotic treatment for elderly patients outside the hospital setting. Although DOACs are considered convenient in these situations, studies on their safety have produced varying results.

### Thromboprophylaxis and treatment of VTE in obese patients

Obesity is increasing rapidly around the world, and it presents a significant health burden [68]. Adult obesity is classified into three categories: class I obesity is defined by a body mass index (BMI) of 30.0–34.9 kg/m^2^; class II obesity by a BMI of 35.0–39.9 kg/m^2^; and class III obesity, or severe obesity, by a BMI ≥40.0 kg/m^2^ [69]. Weight is a VTE risk factor when a BMI exceeds 30 kg/m^2^ [68], and BMI has a strong linear relationship with the incidence of VTE [4]. The inflammatory and metabolic perturbations associated with obesity are thought to provoke a hypercoagulability state in these patients, and central obesity plus high fibrinogen levels may be considered as clinical markers [70]. Genetically pre-determined elevated BMI is associated with a 57% higher risk of VTE (odds ratio 1.57; 95% confidence interval 1.08–1.97; *p* = 0.003), as shown by Mendelian randomisation analysis between BMI- and VTE-associated genetic variants [71]. Presence of other risk factors of VTE, including hospitalisation, pregnancy and use of combined oestrogen–progestin hormonal contraceptives, increases VTE risk in obese patients and exacerbates the severity of VTE [72–74]. An association between elevated BMI and VTE risk has also been recently identified in paediatric patients [75]. Despite greater understanding, a number of questions remain unanswered concerning a definition of high-risk subpopulations who are obese and who may benefit from thromboprophylaxis, the choice of anticoagulants and selection of optimal regimens for thromboprophylaxis, and treatment of VTE in obese patients.

#### Guideline recommendations for obese patients

Clinical practice guidelines such as, National Institute for Health and Care Excellence (NICE), ASH and International Society of Thrombosis and Haemostasis highlight the need for further research regarding dosing regimens for obese patients [14, 76, 77]. Indeed, the experts interviewed considered that prophylaxis and treatment for obese patients should be stratified into subgroups but this is not fully reflected in current guidelines (Table 7).

**Table 7:** High-risk obese patient subgroups that may require variations of VTE treatment

Question	Expert opinion	Guideline recommendations
Do considerations for treatment of obese patients at high risk of VTE vary between patient subgroups?	• Subgroups in obese patients are poorly studied • Treatments vary between different patient weight groups: obese, morbidly obese • The subgroup of obese patients > 120 kg is problematic • Different weight groups require different anticoagulant treatments • Standardised treatment regimens with enoxaparin exist in some hospitals • Medical and surgical obese patients need to be considered as two separate groups • Bariatric surgery or non-bariatric surgery patients and medical patients should be considered separately • Surgical obese patients should be differentiated into those undergoing bariatric surgery or any other surgery • There are differences in how these patients are defined as high risk	ACCP [28]: • Graduated compression stockings are recommended for severely obese patients considering long distance travel ISTH [76]: • Standard dosing of DOACs is recommended for obese patients with a weight < 120 kg • DOACs should not be used in obese patients with a weight > 120 kg but if they are then drug-specific peak and trough levels should be checked NICE [38]: • Further research is needed regarding dose strategies of LMWH for very obese people (BMI > 35) who are admitted to hospital or receiving day procedures • Mechanical prophylaxis is recommended for patients undergoing bariatric surgery RCOG [13]: • Risk of VTE during pregnancy increases with a BMI > 25 and ante-partum immobilisation SOGC [30]: • Recommended dose increases for UFH, enoxaparin, dalteparin and tinzaparin are indicated for obese pregnant women Thrombosis Canada [78]: • Obese patients between 40–100 kg are recommended higher doses of dalteparin, enoxaparin and tinzaparin than patients < 40 kg to be taken once daily. This dose is increased to twice daily for those weighing 101–120 kg

ACCP, American College of Chest Physicians; BMI, body mass index; DOAC, direct oral anticoagulant; ISTH, International Society of Thrombosis and Haemostasis; LMWH, low-molecular-weight heparin; NICE, National Institute for Health and Care Excellence; RCOG, Royal College of Obstetricians and Gynaecologists; SOGC, Society of Obstetricians and Gynaecologists of Canada; UFH, unfractionated heparin; VTE, venous thromboembolism

In certain specific patient subpopulations, such as pregnant women with a BMI ≥40, guidelines suggest prophylactic LMWH dosage appropriate to a patient’s weight should be considered [13], whereas bariatric surgery patients should be given a higher dose of LMWH in combination with graduated elastic compression stockings or intermittent pneumatic compression devices [79]. It is also suggested to avoid dose capping of LMWH, especially in patients with cancer, and to administer LMWH as a twice-daily regimen to allow an adequate total dose to be administered [78, 80].

Due to limited published data on the safety of DOACs, guidelines do not recommend DOACs in patients with a BMI > 40 kg/m^2^ or > 120 kg, and pharmacokinetic/pharmacodynamic data suggest that drug exposure, the peak concentration and half-life of DOACs can be compromised by obesity, leading to underexposure in severely obese patients [76].

#### Anticoagulants, doses and regimens in prophylaxis

**Fig. 1:**
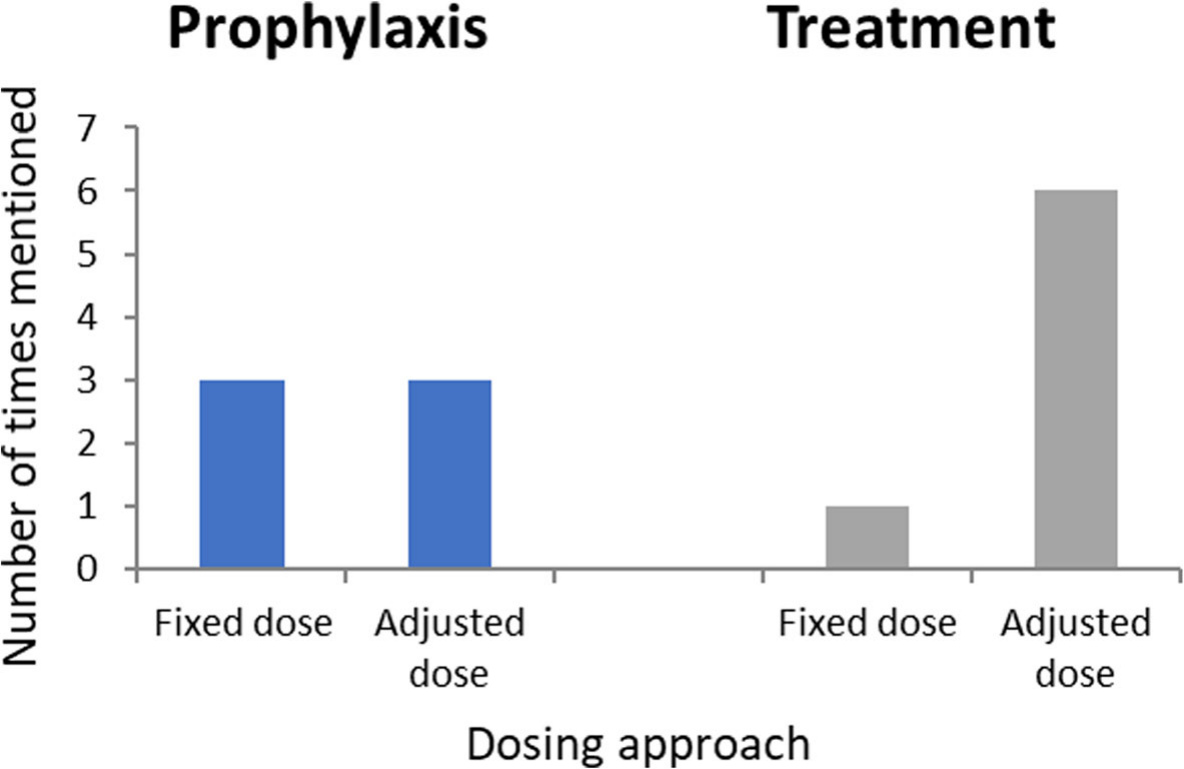
Should weight-based or fixed dosing be used for prophylaxis and treatment of VTE?

The evidence for using anticoagulant thromboprophylaxis in obese patients is scarce according to the experts, as obese (BMI > 30 kg/m^2^) and severely obese (BMI > 40 kg/m^2^) patients are under-represented in clinical studies. Despite this, the physicians interviewed prefer LMWHs over DOACs for thromboprophylaxis of obese patients. A recent pooled data analysis from 11 out of 14 primary studies highlighted the advantages of weight-based or higher-than-fixed dosing of enoxaparin, which increased the probability of achieving desired anti-Xa levels [81]. However, due to insufficient evidence and quality of studies on LMWH dose adjustment, caution should be taken in patients with a weight > 120–125 kg [81]. Indeed, in the UK, NICE have highlighted the need for further research regarding dose strategies for obese patients before recommendations can be made [56]. In Israel, the experts indicated that weight-adjusted regimens are mostly used for thromboprophylaxis in obese patients, and the Canadian experts noted that empiric LMWH dose regimens based on a patient’s weight have been introduced in some hospitals in Canada: patients < 40 kg are given a reduced dose of LMWH (e.g., enoxaparin 30 mg daily) whereas patients > 100 kg receive a higher dose of LMWH, typically increased by 50% (e.g., enoxaparin 60 mg daily or dalteparin 7500 international units [IU] daily). With the latter option, however, they suggest a dose limit should be introduced to avoid overtreatment. The type of LMWH is important, as clearance of different LMWH varies in obese patients. Qualitative interviews revealed divergence of country-specific clinical practices and clinicians’ opinions regarding adjustment of prophylactic LMWH doses to a patient’s weight or BMI (Fig. 1).

Several recent studies have demonstrated advantages of adjusting prophylactic dosage to a patient’s weight to achieve adequate VTE control. High-dose UFH, 7500 IU three-times daily, or enoxaparin, 40 mg twice daily, were more effective in reducing the risk for VTE from 1.48 to 0.77% in patients > 100 kg and discharged from hospital than low-dose UFH (5000 IU twice/three-times daily) or enoxaparin (40 mg once daily), with no increase in bleeding being reported [82]. Similarly, the comparative ITOHENOX study, which evaluated two enoxaparin regimens (60 mg versus 40 mg) in acutely ill obese patients with a BMI ≥30 kg/m^2^, found normal anti-Xa levels in 69 and 31% of patients receiving 60 mg and 40 mg daily, respectively, with no significant difference in bleeding rates between the two groups [83]. Patients with an average BMI of 62.1 kg/m^2^ achieved adequate goal peak anti-Xa levels more frequently when weight-based higher-dose enoxaparin (0.5 mg/kg) was administered compared with a weight-adjusted lower-dose (0.4 mg/kg) or fixed-dose (40 mg daily) regimen [84].

Weight-based enoxaparin dosage for prophylaxis appears more effective than BMI-stratified dosing in achieving anti-Xa levels that are presumed adequate for VTE prophylaxis in severely obese women (BMI ≥40 kg/m^2^) after caesarean delivery [80]. Similarly, another study has shown that post-caesarean weight-based thromboprophylaxis with enoxaparin at 0.5 mg/kg twice daily in women with a BMI ≥35 kg/m^2^ is more effective than fixed dosage of 40 mg daily in achieving prophylactic anti-Xa levels [85].

Efficacy and safety of DOACs in thromboprophylaxis of obese patients has not been adequately investigated. Various Phase III studies of DOACs have a subpopulation of obese patients, but many of those studies are inconsistent in their design, and stratification based on BMI or weight is not always available [79]. Fixed-dose DOACs are generally thought to be inappropriate for patients with a high BMI, specifically a BMI in the range of 30–40 kg/m^2^ and in severely obese with a BMI > 40 kg/m^2^ [80].

**Fig. 2:**
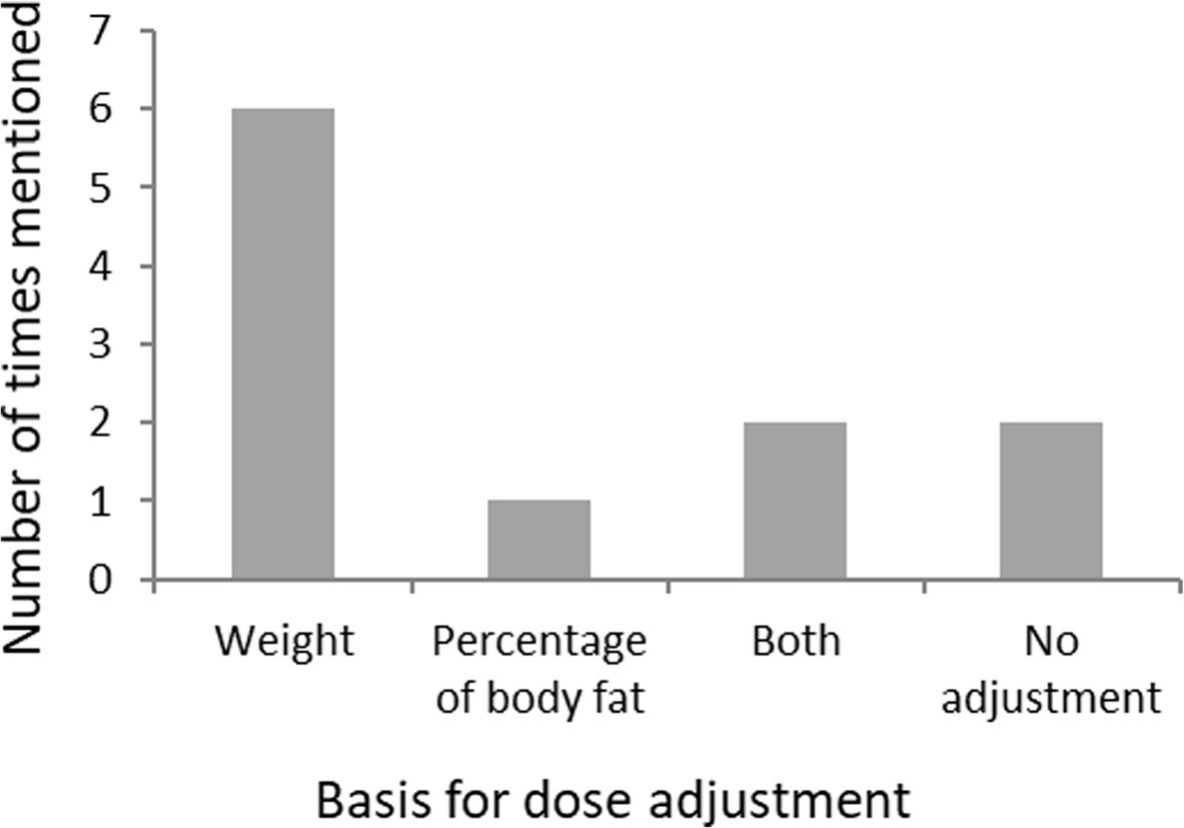
Should dose adjustment be based on weight or related to percentage of body fat?

In summary, due to conflicting data from a small amount of research-based studies in this population, it is uncertain whether dose adjustment should be based on weight, BMI or a fixed-dose regimen (Fig. 2).

#### Treatment of VTE in obese patients

The majority of experts from the qualitative interviews agree on adjusting LMWH dose to a patient’s weight for treatment of VTE (Fig. 1).

In obese patients with a weight > 100 kg and acute VTE, twice-daily dosing with LMWH is suggested. In selected patients, measurement of peak anti-factor Xa levels may be appropriate to ensure that an adequate anticoagulant effect is attained. Therapeutic levels of anticoagulant effect are not established with LMWH therapy and do not appear to correlate with treatment efficacy (VTE recurrence risk) or safety (bleeding risk) [86]. Nonetheless, target peak therapeutic levels have been suggested to be 0.6–1.0 IU/mL for obese patients receiving a twice-daily treatment dose of LMWH and > 1.0 IU/mL for patients receiving once-daily LMWH [86–88].

Recent studies on DOACs showed similar efficacy and safety to that of VKAs in patients with high, normal and low body weight and acute VTE, with similar rates of bleeding episodes recorded [89]. A study by Ihaddadene, et al., and the experts’ personal clinical experience suggest that DOACs, such as rivaroxaban, at a fixed dose is effective in patients with a weight range of 50–150 kg [90]. Moreover, analysis of prospectively collected non-interventional data in stroke prevention in patients with a BMI range of 13.7–57.2 kg/m^2^ and atrial fibrillation or VTE revealed that obese patients treated with a standard dose of DOACs had the lowest rate of cardiovascular events, major bleeding events and all-cause mortality than the normal-weight patients, suggesting that fixed-dose DOACs may provide a safe option in obese patients [91]. Creatinine clearance is greater in obese patients; therefore, it may be suggested to use DOACs with less dependence on renal clearance, such as apixaban or rivaroxaban, in these patients [92]. However, the experts interviewed agreed that further evidence needs to be generated to recommend DOACs for obese patients in a routine clinical setting.

#### Anticoagulants and dose regimens in bariatric surgery

The number of patients undergoing bariatric surgery procedures is increasing, and VTE prevention research in this area warrants more attention to define best practices. Patients undergoing bariatric surgery are considered at high VTE risk due, in part, to such patients having multiple non-surgical factors that increase risk, and VTE is also likely one of the most common causes of death in this population [93, 94]. In the US, bariatric surgery is the most common surgery [95]. However, studies carried out on patients undergoing bariatric surgery do not necessarily reflect up-to-date practices according to the experts, as patients are currently discharged from hospital 1–2 days post-surgery and therefore thromboprophylaxis should be considered in an out-of-hospital setting. Evidence-based guidance is sparse, but it suggests that the LMWH dose should be increased for prophylaxis, with a weight-based or staggered dose, after bariatric surgery [39]. Indeed, a systematic review, which sought to discover if weight-adjusted thromboprophylaxis is safe and effective in the post-operative period, showed that prophylactic doses of heparin, adjusted to a patient’s weight, achieved a significantly better reduction in the in-hospital VTE rate when compared with non-adjusted prophylactic dose (0.54% versus 2.0%) [94]. A risk-assessment tool was designed to predict the risk of post-discharge VTE, which was 0.29% in a 30-day post-bariatric surgery period with a 28-fold increase in mortality in those with VTE (*p* < 0.001) [94]. More than 80% of VTE events occur in a post-hospitalisation period, and this proportion is likely to become larger as bariatric surgery is increasingly done as a day procedure or with a minimal hospital stay [94]. In a prophylactic setting following bariatric surgery, anti-Xa measurements (trough levels if intent is to identify over-dosing and peak levels if intent is to identify under-dosing) should be considered 3–5 days after starting prophylaxis, but patients typically are sent home 1–2 days post-operatively and are in the acute-phase setting, so routinely measuring anti-Xa levels may be impractical. The experts considered that the determination of VTE is typically made pre-procedure, so anti-Xa levels appear uninformative in stratifying patients for prophylaxis.

## Conclusions

The findings from the interviews with experts, the Thrombosis Think Tank meeting and the desktop research highlight the inconsistency of guideline recommendations and the heterogeneous views of physicians on effective primary and secondary VTE prophylaxis and VTE prevention in these high-risk medical patients. There is a paucity of user-friendly, population-adapted VTE risk-assessment models to provide reliable stratification of medical patients for anticoagulant therapy, and current biomarkers show promise when investigated in research studies, but many have little value in the routine clinical setting [43].

LMWH remains the anticoagulant of choice in pregnant women and obese patients, where DOACs are not currently recommended. Similarly, in the elderly, LMWH demonstrates a better safety and efficacy profile than UFH for thromboprophylaxis. LMWH dose adjustment remains a significant problem in obese patients and pregnant women, with conflicting views on adjustment of prophylactic dose related to weight or BMI. Simple guidance needs to be generated for clinicians, as many are not familiar with the use of pharmacokinetic data to adjust dosing regimens.

Although further clinical studies are needed to address the VTE prophylaxis gaps, ultimately, global communication on best-practice strategies and homogeneity of guideline recommendations through increasing research data would help join up the gaps in clinical practice and improve the outcomes of medical patients.

## Supplementary information

**Additional file 1.** Qualitative interview questionnaire.

**Additional file 2.** Author video evaluating unmet clinical needs in prophylaxis and treatment of venous thromboembolism in at-risk patient groups.

## Data Availability

Not applicable.

## Abbreviations

ACCP: American College of Chest Physicians
ASH: American Society of Hematology
BMI: Body mass index
DOAC: Direct oral anticoagulants
DVT: Deep vein thrombosis
IU: International unit
LMWH: Low-molecular-weight heparin
NICE: The National Institute for Health and Care Excellence
PE: Pulmonary embolism
RCOG: Royal College of Gynaecologists
RCT: Randomised controlled trial
UFH: Unfractionated heparin
VKA: Vitamin K antagonists
VTE: Venous thromboembolism
