# Supplementary material for: Evaluation of unmet clinical needs in prophylaxis and treatment of venous thromboembolism in at-risk patient groups: pregnancy, elderly and obese patients
Source: Thromb J. 2019 Dec 27;17:24. doi: 10.1186/s12959-019-0214-8 (PMC6935082; doi:10.1186/s12959-019-0214-8)
Supplement: Supplementary file 1 — Additional file 1. Qualitative interview questionnaire. [file 12959_2019_214_MOESM1_ESM.docx]

Additional file 1

*Qualitative interview questionnaire*

| **Question:** |
| --- |
| **PART ONE** |
| **Personal:** |
| What do you consider to be your clinical speciality? |
| What is your main area of interest relating to thrombosis and anticoagulant therapy? |
| **PART TWO (appropriate sections of PART TWO will be selected depending on expertise and response to earlier questions)** |
| **PART TWO (a)** |
| **Pregnant women at high-vascular and VTE risk** |
| What guidelines and clinical protocols do you use for prevention and treatment of venous thromboembolism (VTE), including guidance on dose and duration, in ante- or post-partum pregnant women? |
| What guidelines and clinical protocols do you use for prevention of VTE, including guidance on dose and duration, in women with recurrent pregnancy loss? |
| What method do you use to identify optimal dose of anticoagulants in thrombophilic pregnant women and those with pregnancy loss, e.g., pharmacokinetic/pharmacodynamic (PK/PD) modelling or other methods? |
| Which subpopulation(s) of pregnant women, ante- or post-partum, or those with recurrent pregnancy loss, should be treated with low-molecular-weight heparins (LMWH) such as enoxaparin? |
| Where do biosimilars of LMWHs fit into the treatment of pregnant women? |

| **PART TWO (b)** |
| --- |
| **Elderly** |
| Do you think there is sufficient evidence to demonstrate that current practices for treating VTE are effective in elderly patients? |
| Are there any practical considerations when treating elderly patients with high risk of VTE, such as specific risk factors, contra-indications, comorbidities or practicalities of administration? |
| In which subgroups of elderly patients would you consider LMWHs, such as enoxaparin, the optimal choice? |
| Should extended prophylaxis be used in elderly patients, e.g., for hip fractures? |
| Where do biosimilars of LMWHs fit into the treatment of elderly patients? |
| **PART TWO (c)** |
| **Pharmacologic modelling in obese patients at high risk of VTE** |
| Do considerations for treatment of obese patients at high risk of VTE vary between patient subgroups? |
| Are there any specific challenges or considerations for prevention and treatment of VTE during bariatric surgery? |
| What guidance do you use to determine the dose of anticoagulants in obese patients, e.g., for knee or bariatric surgery; are further support tools or evidence required? |
| Should dose adjustments be made based on weight or related to percentage of body fat? |
| Should a weight-based dosing approach be used for treating VTE and a fixed dose for prophylaxis? |
| Do you think PK/PD modelling could be used to identify optimal dose of anticoagulants in obese patients? |
